# Validation of food frequency questionnaire for food intake of adults in Gida, West, Ethiopia

**DOI:** 10.3389/fpubh.2024.1438008

**Published:** 2024-11-28

**Authors:** Worku Fikadu, Segni Mulugeta, Yeshimebet Dejene

**Affiliations:** ^1^Department of Public Health, Institute of Health Science, Wollega University, Nekemt, Ethiopia; ^2^Saint Peter Specialized Hospital, Addis Ababa, Ethiopia

**Keywords:** food frequency questionnaires, Gida Woreda, 24-hour dietary recall, validity, Ethiopia

## Abstract

**Background:**

Diet is characterized by complex exposure to strongly intercorrelated components. Early efforts to understand diet-disease associations focused on the role of specific nutrients, but later, it became evident that dietary exposures may act synergistically in several instances. For research into how nutrition affects health and disease, scientifically sound descriptions of dietary intake at the population level are essential. Although food frequency questionnaires are important nutritional assessment methods, they should be validated and checked for reliability according to the eating habits of the specific population. Context-specific tools are needed to estimate food intake accurately, but a single study in Ethiopia has not established reliability. Hence, this study aimed to establish a valid and feasible dietary assessment method for 24-h dietary recall.

**Objective:**

To adapt and validate FFQs for use as dietary assessment tools in epidemiological studies among adults in Gida woreda, West Ethiopia.

**Method:**

A community-based cross-sectional study was conducted from February 15–30, 2023, among 150 participants. Data were collected through an interviewer-administered questionnaire, a focus group discussion, and a key informant interview. We compared the mean of three interactive 24-h dietary recalls to the FFQ to assess its relative validity. By comparing food category levels, mean differences, medians, and cross-classifications from the FFQ and a 24-h dietary recall, validity was assessed.

**Results:**

The response rate of this study was 100% for both the FFQ and 24-item dietary recall. The mean (±SD) age of the participants was 37.6 9.7 years. Of the total participants, 40 (29%) were between 31 and 35 years old. Sixty-four (42%) of the study participants were protestant religious followers. The median ranges from zero for meat and poultry to 1,930 for cereal, as estimated by the 24-h dietary recall method. The median ranges from 14 mg/day for meat and poultry to 724 mg/day for cereal, as estimated by the FFQ.

**Conclusion:**

This study revealed that this food frequency questionnaire had good validity for capturing the intake of food groups which indicated by their value: vegetables (0.8), legumes (0.9), roots/tubers (0.8), cereal (0.5), dairy products (0.75), and meat/poultry (0.64) at both the individual and group levels. Hence, it is recommended for health care providers to use FFQ as a tool for studying and managing dietary-related diseases.

## Introduction

A healthy diet helps protect against malnutrition and diet-related noncommunicable diseases such as diabetes, heart disease, stroke, and cancer. A healthy diet should include fruits, vegetables, legumes, nuts, and whole grains, with a daily intake of 400 g. Free sugars should be < 10%, fats should be < 30%, and salt should be < 5 g daily (1). The human body functions optimally when it consumes a variety of food. A diverse diet can reduce the risk of chronic diseases and promote good health. Different dietary patterns affect bodily functions through different mechanisms, influencing the body's response to nutrients. Understanding food and meal-based dietary patterns is crucial for maintaining the health of individuals (2). Accordingly establishing a good and easily applicable measure of frequency of the diet is very important (2, 3).

Eastern Mediterranean Region shows the highest levels of overweight people in Kuwait, Egypt, the United Arab Emirates, Saudi Arabia, Jordan, and Bahrain, with the prevalence of overweight/obesity ranging from 74 to 86% among women and 69%−77% among men (1). the prevalence of obesity and overweight in Ethiopia ranges from 1.7 to 3.6%. Regarding food items, about 75% of the Ethiopian diet is cereal-based monotonous feed. Other studies reveal that the prevalence of low and medium dietary diversity scores among Ethiopian populations was 60 and 40%, respectively (3).

Food frequency questionnaires (FFQs) are a condensed list of foods and drinks that require respondents to indicate how frequently they consumed each item during a predeterperiod (1–3). Food frequency questionnaires (FFQs) are a popular method for assessing dietary patterns and determining the impact of diet on health. These questionnaires are relatively inexpensive and impose fewer burdens on respondents than other dietary assessment methods (4). However, the accuracy and reliability of diet measurements still present an ongoing challenge. Although weighed food records and 24-h recalls have been widely used, their substantial burden on respondents and economic constraints make them inapplicable for most large epidemiological studies (5–7). FFQs provide an overview of typical food or nutrient intake across time, but they may not always represent foods consumed by other populations due to their percentage contribution to overall nutrient consumption among representatives of the target population (8, 9). Education level and interest in diet also affect how accurately people recall meals they have eaten. Earlier studies showed that FFQ tended to overestimate certain foods, such as fruits and vegetables, and underestimate others, such as meat and meat products (10–13).

This validation study evaluated the validity of a food frequency questionnaire for food intake against the 24-h diet recall method among adults in Gida Woreda, West Ethiopia. Despite many food frequency validation studies conducted globally, there are still large differences in the results of these studies. There is underreporting among men and women in terms of energy intake of 12%−14% and 16%−20%, respectively. Factors and behaviors may explain the underreporting of energy intake and macronutrient and nutrient intake in different populations, including physiological, sociodemographic, psychological, and lifestyle attributes (3, 14–18). Repeatability was assessed in only 47% of the validation studies in the review (9). It is not wise to administer a questionnaire at a very short interval, as respondents may remember their previous responses. Alternatively, when longer intervals are used, true changes in dietary habits and variations in responses contribute to reduced reproducibility (19).

Common dietary assessment methods, such as 24-h recall and dietary records, only include current dietary intake data (20, 21). Short-term memory and diet record systems are expensive, unreliable, and unfit for analyzing previous diets. FFQs are effective and reliable tools for assessing mid- to long-term dietary habits, and establishing long-term dietary assessment methods, such as food frequency questionnaires, will improve dietary habits (2, 4, 14). A food-frequency questionnaire's list of food items varies significantly, with a median of 79 items which ranges from 5 to 350 food items (2). Willett suggests that larger and more thorough questionnaires have a decreasing marginal gain, so unnecessary expansion of the list of foods is not beneficial. Excessive questions can become tedious for interviewers and participants, making it unnecessary to include unnecessary questions in a food-frequency questionnaire (2, 14). There were no previous studies in the in-study setting and this study will improve healthy diet measurement and early intervention by health care providers.

## Methods and materials

### Study area

This study was conducted in Gida Woreda. The district is located in western Ethiopia, 441 km away from the capital city of Addis Ababa. It has 28 kebeles and two administrative towns. According to reports from the district, 158,296 of the 79,048 (49.93%) patients were females, and 79,248 (50.06%) were males. The district has one hospital, six health centers, seven urban health posts, 22 rural health posts, and 26 private clinics. There were 2,447 patients younger than 1 year of age, 12,488 younger than 5 years of age, 16,798 children of childbearing age and 2,637 pregnant individuals. Furthermore, there were two health centers and 10 private health institutions delivering health services in the Woreda.

### Study design and period

A community-based cross-sectional study was conducted from February 15–30/2023.

#### Source population

The source population for this study was all adults who were residents of Gida Woreda.

#### Study population

The study population included randomly selected adults who were residents of selected kebeles of Gida Woreda during the data collection.

#### Inclusion criteria

All the adults who were residents of Gida Woreda lived for at least 6 month before the data collection period in the study area.

#### Exclusion criteria

Adults who were disabled (unable to hear) or unable to give verbal consent were excluded.

### Sample size calculation

The sample size was calculated based on the objective to determine the mean difference between the two measurements. Based on the following assumption, the mean difference for consuming vegetables is 5.42 and the standard deviation is 14 and 9, respectively, for the 24-h recall method and FFQ (8).

n $={\left(Z_{\text{α}/2}\;=\;Z_{\text{β}}\right)}^{2}\;*\;2\;*\;\sigma^{2}/d^{2}$ where Z_α/2_ is the critical value of the normal distribution at α/2, *Z*_β_ is the critical value of the normal distribution at β (for a power of 80%, the critical value is 0.84), σ^2^ is the population variance, and d is the difference. The validation research used a sample of 61–408 participants to validate the FFQs (7). The study's design considers factors like population type, subject and replicate days, and diet type. Cade et al. recommend a sample size of at least 50 subjects, with 100 or more ideal for the Bland-Altman approach (18). This sample size is based on the assumption that a sufficient number of day-worth of dietary data is required to accurately depict a person's genuine diet (11–13). The sample size required for a statistical method to assess reproducibility and validity depends on the method used. The review study showed a wide range of sample sizes, from 6 to 3750, with a median of 110. For repeatability studies, a sample size of at least 50, preferably larger (100 or more subjects), is desirable (1, 3, 4). To improve precision, two measurements on each subject by each method are valuable. For the correlation coefficient, a sample size of no more than 100 to 200 should be sufficient, assuming a sufficient number of days of dietary information is obtained to reasonably describe an individual's diet (3, 9, 10). However, few studies manage to obtain such a large number of days of good-quality dietary information from their subjects, so most use between two and five replicates per subject. If a strategy using a small number of replicates per subject is employed, the number of subjects needs to be increased to maintain the same precision of the corrected correlation coefficient (3, 4, 10). The final sample size was determined based on the following assumptions: obtaining a sufficient number of days of dietary information to reasonably describe an individual's diet, which is 14 to 28 days, that enables the Bland-Altman method to determine the validity of the tool by using a sample size of at least 50, and using another validation study that suggests 150–200 sample sizes can add precision (17). Considering the above recommendations, we used 150 study units for this study.

### Sampling technique and procedures

**Figure 1:**
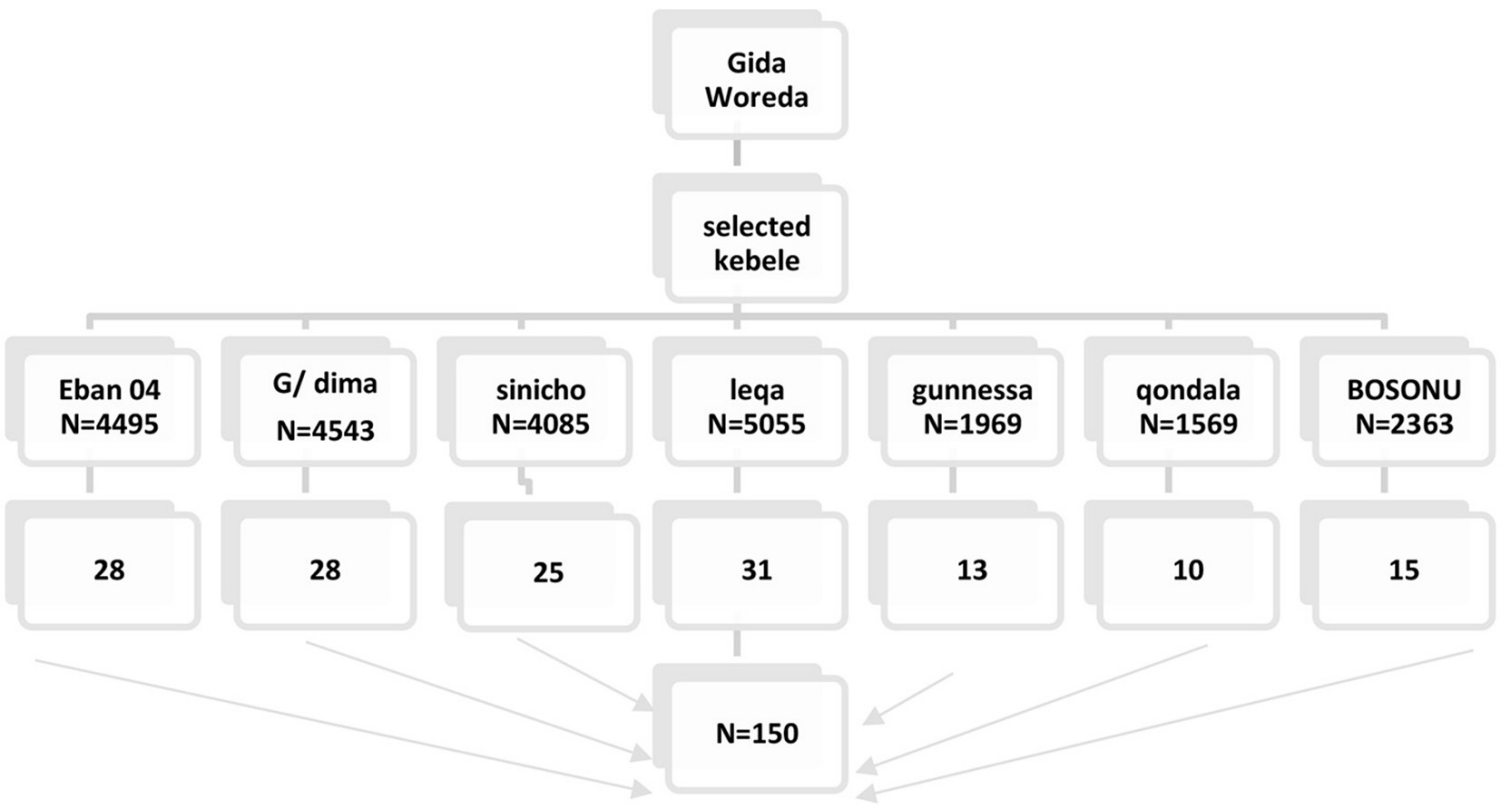
Schematic representation of the sampling procedure for adult Gida Woreda Kebele.

A simple random sampling technique was applied to select the study participants. A total of seven kebeles were included to reach the desired sample size. The sampling frame with the list of households was obtained from each kebele's health post database. The random lists of samples (the number chosen by a computer to represent the study unit) were obtained through a computer random number generator. Then, the generated random numbers were compared to the lists of households in the health post Community Health Information System (CHIS). The final sample size was allocated proportionally to the number of kebeles to identify households in each kebele to be selected (Figure 1).

### Data collection tools

#### Sociodemographic characteristics

A questionnaire adapted from the Ethiopian demographic and health survey (EDHS) was used to obtain sociodemographic data, including age, sex, occupation, educational status, and marital status.

### Food intake assessment

The data collection tool for dietary intake was adapted from previously published material from Butajira for FFQ. Foods that were not included in earlier studies conducted in Butajira were included in the current questionnaire, and foods that were not common in our study area were removed from the current FFQ. Foods incorporated in the current FFQ are Ancotte under roots and tubers sorghum and sorghum with maize under cereal products. Fish and fish products were removed from the current FFQ. Multiple 24-h dietary recall questions adapted and validated for use in developing countries were used as a reference method for the validation of a food frequency questionnaire. The original Butajira study reviewed to identify food items suitable for Gida ayana culture, using nine food groups (8). A preliminary study was conducted to identify additional foods commonly consumed by the district community. The FFQ consisted of 89 food and beverage items, categorized into 9 food groups. The main food groups are cereals and cereal products, meat and meat products, eggs, legumes and its products, milk and dairy products, vegetables, fruits, roots and tubers, and beverages. Each item was rated on intake frequency over the past month, with eight categories: never, 1–3 times per month, once a week, 2–4 times per week, 5–6 times per week, once a day, 2–3 times per day, and ≥4 times per day. Household units were used for each food item, such as a scoop, plate, bowl, cup, and tablespoon.

### Measurement and variables

#### Adapting food frequency questionnaire

The FFQs were adapted through five major steps: a critical review of previous FFQs, 24 h and FGDs to identify new food items and beverages, regrouping and clustering of previous and new items, expert consultation for content validity, and pretesting of the FFQs. Common food items were chosen and prioritized after consultation with the culture and tourism office of Gida Woreda. Focused group discussions and 24-h dietary recall were conducted among 10 women living in Gida Woreda to identify and prioritize common diets. Ancotte and sorghum were common, while fish and fish products were not common in the area. Similar food items and beverages were regrouped and clustered into previous and new categories. A new portion size estimation method was used for new categories, and the frequency of intake was evaluated based on the usual intake over the previous month. A prespecified portion size estimation method was employed for the FFQ. Experts reviewed the newly adapted FFQ to confirm its content validity. The food list was extensively discussed, and pretesting was conducted with 10 adult women from a nearby site. Some minor changes will be made as a result.

### Data collection procedures

#### Recruiting data collectors

Three interviewers who had previous experience in dietary data collection and who were fluent in the local language were recruited. The data collectors were trained for five days on how to conduct the FFQ and 24-h diet recall interviews. For the interactive 24-h recall, on day 1, the purpose of the interactive 24-h recall was explained, and working arrangements were discussed. On Day 2, details on how to conduct the 24-h recall interviews and any associated questionnaires were discussed, with some interviews on recording and describing the foods and drinks consumed using hypothetical menus. Days 3 and 4 focused on methods to estimate portion sizes, how to complete the 3 passes of the interview protocol correctly, and how to practice a 24-h recall interview among themselves. On Day 5, instructions were given on how to complete the recipe forms and how to handle difficult scenarios, and a field exercise was conducted. The participants also received training on how to administer the FFQ. The frequency of consumption and portion size estimation issues and issues relevant to FFQ data collection were discussed during training. Corrections and feedback were given accordingly.

### Dietary intake assessment

The FFQ was validated against the average of three unannotated 24-h recall questionnaires. The FFQ score was obtained after the second 24 hr of diet recall. There was a 15-day interval between the first 24 hr of diet recall, the second 24 hr of diet recall and the third 24 hr of diet recall.

### 24-h dietary recall

The Food Frequency Questionnaire (FFQ) is validated using 24-h dietary recall, which is less time-consuming and less likely to impact dietary habits. However, it has been criticized for overestimating actual intake (14–18). Direct records are suggested as an ideal comparison approach, but their application is limited by dietary changes and the need for highly motivated, numerate, and literate participants (19–21). Multiple 24-h dietary recalls were collected and used as a comparison standard for validation.

We conducted three 24HRs on nonconsecutive days on 8 different food groups. The diet recall test used was an interactive, multiple-pass 24-h dietary recall test adapted and validated for use in developing countries. The multiple-pass technique was used after rigorous training, and the pretest was conducted before dietary data collection. Each interview involved a stepwise series of questions, and common household utensils such as bows, plates, spoons of different sizes (tablespoon, teaspoon), coffee cups, teacups, and water glasses were used to improve the memory of the respondents and to assist in completing the recall. The interviews were conducted on weekdays, fasting days and weekends to capture variance in intake across various days of the week.

### Food frequency questionnaire

The frequency categories for the questionnaire should be continuous, with 1–8 choices, emphasizing the more frequent end for most foods and a less frequent option for infrequently eaten foods (1, 8, 9). The frequency of intake was evaluated based on the usual intake over the previous month for 8 different groups. Some validation studies administered the FFQ both after and before the study, while other studies administered the FFQ once, either before or after the reference method (8, 11). To reduce any bias introduced by using only the first or second FFQ, an additional option would be to randomly use either the participant's first or second FFQ data for comparison with the reference method. This approach would provide a combination of the minimal and maximal validities (1, 10). Validation studies suggested 24 DRs three times (1 week day, one week of fasting, and one week of weekend day) to incorporate different eating habits. The FFQs will be provided for the same participant to complete the first round of the 24DR. The same participant who had completed the 24DR was asked to complete the FFQs. Participants were asked to provide detailed descriptions of the food and beverages consumed, the food preparation method, and the brand of the food and beverages consumed (8). Nine frequency categories were included, ranging from daily to never/one less than per month, and each food item was assigned a portion size.

Portion size estimation: A prespecified portion size estimation from dietary guidelines based on average consumption was employed for the estimation of portion size using local household units such as bowls, plates, spoons of different sizes (tablespoon, teaspoon), coffee cups, teacups, water glasses, and photographs. A visit to local markets and shops will be held to purchase and arrange equipment for data collection. To help standardize participants' understanding, the interviewer prepared photographs for each measurement and showed them to the participants. First, the participants were asked to report whether they consumed each food item. If they answered yes, they were asked to indicate how many times per day, per week, and per month. When they are asked about their usual portion size, they will be asked to report the average quantity or portion of the food item on the day of consumption, which includes totals of all meals (breakfast, lunch, snacks, dinner), expressed as common household measures such as a ladle, small cup or spoon.

### Data quality assurance

The data collectors and field supervisors provided rigorous training on FFQs and 24-h diet recall for five days. The purpose of the data collection will be explained in detail. Immediately after the interview was conducted, both the investigator and the data collectors rechecked the questionnaire for completeness and consistency. The questionnaires were also reviewed to ensure that they were neat and legible, that all the information was properly collected and recorded, and that no information was missing. A pretest will also be conducted.

### Statistical analysis

All the statistical analyses were performed using IBM^®^ SPSS Statistics version 23 (Chicago, IL, USA). Before the statistical tests were carried out, the normality of the data in this study was tested using the Shapiro–Wilk test with a 0.05 significance level. Descriptive analysis was used to obtain the frequency, percentage, mean, and standard deviation of sociodemographic, anthropometric, and dietary intake data. Descriptive statistics of daily food intake calculated from the FFQ and 24DR are presented as the mean values ± standard deviations (SD). Wilcoxon signed-rank tests were used to compare values obtained from the FFQ and 24DR and values obtained from the FFQ1 and FFQ2, as the data were not normally distributed.

### Validity assessment

The validation research used a sample of 61 to 408 participants to validate the FFQs. The study's design considers factors like population type, subject and replicate days, and diet type. Cade et al. recommend a sample size of at least 50 subjects, with 100 or more ideal for the Bland-Altman approach (7, 18). This sample size is based on the assumption that a sufficient number of day-worth of dietary data is required to accurately depict a person's genuine diet.

For the validity assessment of the FFQ, food intake derived from the FFQ was compared with that derived from the 24DR using Spearman's correlation coefficient. In addition, the Bland–Altman plot was used to graphically examine the agreement between the FFQ and 24DR for food intake. The cutoff points that were used for the correlation coefficient were as follows: < 0.20 for low correlation, 0.20–0.49 for moderate correlation and ≥0.50 for high correlation. Bland–Altman plots show the differences in intake between the two methods (FFQ – 24DR; *y*-axis) against the mean intake of the two measures [(FFQ + 24DR)/2; *x*-axis]. This was done in SPSS, and since we do not have a direct Bland–Altman plot in SPSS, we followed five steps to construct the plot. Step 1: calculate the difference of the two measurement for each food category, step 2: compute the mean of difference for each food category, step 3: compute the standard deviation for each food category and multiply 1.9, step 4: compute 95% limits of agreement *Mean difference*±1.96 × *standard deviation*, step 5: construct scatter plot with reference line on *y*-axis by using mean difference, the upper and lower 95% limit of agreement. Across-classification of quartile analysis was used to examine agreement between the two tools in terms of proportion of participants' food intakes. Cross-classification analysis will be performed by classifying participants into quartile categories based on the dietary intake data from both the FFQ and 24DR. This was done after identifying the first quartile data point from the 24-h dietary recall method and counting the data points below/above and dividing by the denominator (number of participants).

## Results

### Sociodemographic characteristics of the study participants

A total of 150 study participants were included in the study, with a response rate of 100% for both the FFQ and 24-day dietary recall. The mean (±SD) age of the participants was 37.6 ± 9.7 years. Of the total participants, 40 (29%) were between 31 and 35 years old. Sixty-four (42%) of the study participants were protestant religious followers. One-fifth (20%) of the participants attended primary education. Of the total participants in the study, 40% (60) were farmers (Table 1).

**Table 1:** Sociodemographic characteristics of the adults in Gida Woreda, West, Ethiopia in 2023.

		**Frequency**	**Percent %**
Age-group	18–25	21	14.0%
	26–30	11	7.3%
	31–35	44	29.3%
	36–40	14	9.3%
	41–45	31	20.7%
	>46	29	19.3%
Religion	Orthodox	60	40.0%
	Muslim	17	12.3%
	Protestant	64	42.7%
	Other^**^	9	5.0%
Education level	Primary (1–8 grade)	25	20.8%
	Secondary (9–10)	28	23.3%
	Technical	33	27.5%
Occupation	Government/ private employee	54	36.2%
	Merchant	35	23.5%
	Daily laborer/farmer	60	40.3%

^**^Others: (Wakefata, Catholic, Adventist).

The mean (SD), median, and 25th and 75th percentiles of daily food group intake.

The median and 25th and 75th percentiles of daily food intake were estimated by the 24-h dietary recall test and FFQ. The median ranges from zero for meat and poultry to 1,930 for cereal and cereal products, as estimated by the 24-h dietary recall method. The median ranges from 14 mg/day for meat and poultry to 724 mg/day for cereal and cereal products, as estimated by a food frequency questionnaire. The 24-HR dietary recalls and FFQs yielded similar median intake estimates for legumes, roots/tubers, dairy products and eggs. For fruits, vegetables and meat/poultry, the FFQ yielded a greater median estimated intake than did the 24-h dietary recall. The Wilcoxon signed rank test for related samples was conducted to establish significant differences in the medians. For cereal and cereal products, vegetables and beverages, there were statistically significant differences in the medians (Table 2).

**Table 2:** Wilcoxon signed rank test for daily food group intake among adults in Gida Woreda, West Ethiopia, 2023.

	**24-HR diet recalls**		**Food frequency questionnaire**		
Food types	Median	(25%, 75%)	Median	(25%, 75%)	Wilcoxon signed Rank test
Cereal	1,930 g/day	(1,817, 2,159 g/day)	727 g/day	(672, 799)	0.000^**^
Roots and tubers	324 g/day	(255, 369 g/day)	324 g/day	269, 379 g/day	0.470
legumes	334 g/day	(25, 334 g/day)	334 g/day	100, 535 g/day	0.535
fruits	150 g/day	(0, 150 g/day)	200 g/day	(150 g/day, 600 g)	0.056
vegetable	135 g/day	(106, 135 g/day)	171 g/day	(114, 394 g/day)	0.042^*^
Dairy products	200 g/day	(0, 200 g/day)	200 g/day	(0, 400 g)	0.9 00
Eggs	50 g/day	(50 g−100 g)	50 g/day	(0, 50 g/day)	0.91
Meat and poultry	10 g/day	(0, 103 g/day)	14 g/day	(0, 154 g/day)	0.700
Beverages	0	(0, 0)	576	(288, 728 L/day)	0.000^**^

^*^p value ≤ 0.05.

^**^p value < 0.01.

### Correlations between food group intake

After checking for monotonic correlation of the variable, the Spearman correlation coefficient was calculated to determine the strength of the relationship between the two measures. Spearman correlation coefficients ranged from 0.02 for vegetables to 0.85 for roots and tubers, and a correlation coefficient >0.5 was observed for legumes (*r* = 0.75). Correlations (*r* = 0.4) were detected for cereals, meat/poultry/fish (*r* = 0.05), fruits (*r* = 0.43), dairy products (*r* = 0.23), roots and tubers (*r* = 0.85), vegetables (*r* = 0.23) and beverages (*r* = 0.65). The correlation was low (< 0.2) for eggs (*r* = 0.74). Except for meat/poultry and beverage intake, all correlations were statistically significant (Table 3).

**Table 3:** Correlations between food group intake obtained from the 24-HRs and the FFQ among adults in Gida Woreda, west Ethiopia.

**Food group**	**Mean difference**	**95% limit of agreement**	**Spearman**
Cereal and its products	−93	(−497, 311.7)	0.401^*^
Vegetable	−283	(−751.4, 185.4)	0.02^*^
Legumes	315	(−61.25, 713)	0.75
Eggs	49.9	(−13.5, 113.5)	0.74^**^
Roots and tubers	266	(−807, 277)	0.85^**^
Dairy products	7.3	(2.49, 11.5)	0.23^**^
Fruits	−384	(−1,203, 435.2)	0.43^*^
Meat and poultry	58	(−64.5, 180.5)	0.05
Beverages	0.9	(−0.84, 2.6)	0.65

^*^p value ≤ 0.05.

^**^p value < 0.01.

### Agreement of FFQ and 24 h dietary recall on classifying food intake into categories

The proportion of individuals classified by the FFQ and the average of two 24-h dietary recalls into the same quartile ranged from 2% for beverages to 61.3% for legumes. However, the proportion classified into opposite quartiles varied from 2% for cereal and cereal products to 24% for beverages. No gross misclassification was observed for the intake of eggs. The weighted kappa values ranged from 0.33 for eggs to 0.9 for legumes (Table 4).

**Table 4:** Cross-classification based on quartile and weighted kappa statistics of daily food intake between FFQ and 24HR recall dietary method among adults in Gida Woreda West Ethiopia in 2023.

**Food groups**	**Cross-classification of based on the quartile**		**Weighted Kappa Good >0.6**
	**Proportion of individuals in the same quartile**	**Proportion of quartile individuals in the opposite**	**Value for kappa**
Cereal and its products	29%	2%	0.50
Vegetable	42%	6.4%	0.80
Legumes	61.3%	11.3%	0.90
Eggs	54%	0%	0.70
Roots and tubers	46.2%	4%	0.80
Dairy products	38%	7.6%	0.75
Fruits	19%	4.7%	0.33
Meat and poultry	33.9%	14%	0.64
Beverages	2%	24%	0.02

The one sample *t*-test showed that the means of the differences for cereal and cereal products, legumes, vegetables, dairy products, eggs, roots/tubers, meat/poultry, beverages and fruits did not differ from zero between the two measurements of food intake. This shows that the FFQ and 24-h recall methods measure the same intake amount for these food groups (Table 5).

**Table 5:** One sample *t*-test for the difference between 24-h recall and FFQ methods for food intake among adults in Gida Woreda, western Ethiopia, in 2023.

**One-sample test**						
	**Test value** = **0**					
	***t***	**df**	**Sig. (2-tailed)**	**Mean difference**	**95% confidence interval of the difference**	
					**Lower**	**Upper**
Cereal and its products	1.000	149	0.319	−93	−497	311.7
Legumes	2.312	149	0.238	315	−61.25	713
vegetables	1.991	149	0.789	−283	−751.4	185.4
Dairy products	3.003	149	0.555	7.3	2.49	11.5
Eggs	1.222	149	0.899	49.89	−13.5	113.5
Roots and tubers	0.009	149	0.993	266	−807	277
Meat and poultry	0.177	149	0.344	58	−64.5	180.5
Beverages	1.200	149	0.086	0.9	−0.84	2.6
Fruits	2.030	149	0.075	−384	−1,203	435.2

p < 0.05 indicates that the difference is not zero.

Linear regression revealed that the coefficients of difference for cereal and cereal products, legumes, vegetables, dairy products, eggs, roots/tubers, meat/poultry, beverages and fruits did not differ from zero between the two measurements of food intake. This shows that there is no proportional bias in measuring food intake above or below the mean of the difference for these two measurements of food intake (FFQ and 24-h recall methods; Table 6).

**Table 6:** Linear regression for the difference between FFQ and 24HR among adults in Gida Woreda, western Ethiopia, in 2023.

**Model**		**Unstandardized coefficients**		**Standardized coefficients**	***t***	**Sig**.
		***B***	**SE**	**Beta**		
1	DIFF for cereal between FFQ and 24 h recall	−0.002	0.012	−0.013	−0.157	0.875
2	DIFF for meat/poultry between FFQ and 24 h recall	1.22	0.345	2.44	0.022	0.65
3	DIFF for root/tubersl between FFQ and 24 h recall	−1.007	0.024	−1.89	−0.456	0.966
4	DIFF for fruits between FFQ and 24 h recall	−3.00	−0.77	−0.655	2.067	0.598
5	DIFF for vegetables between FFQ and 24 h recall	0.23	0.788	1.456	0.799	0.82
6	DIFF for dairy products between FFQ and 24 h recall	0.923	0.66	0.422	0.1.77	0.98
7	DIFF for beverages between FFQ and 24 h recall	−0.098	0.85	−0.66	−0.354	0.601
8	DIFF for legumes between FFQ and 24 h recall	0.66	0.006	0.22	0.055	0.804
9	DIFF for eggs between FFQ and 24 h recall	−0.562	0.87	0.446	0.243	0.9

p < 0.05 indicates the presence of proportional bias.

### Bland–Altman analysis

A systematic trend of overestimation for roots and tubers and underestimation of beverage intake at higher values was observed when we used the FFQ. The majority of the data points are within the 95% limits of agreement for almost all food groups. A wide limit of agreement was observed for fruits.

**Figure 2:**
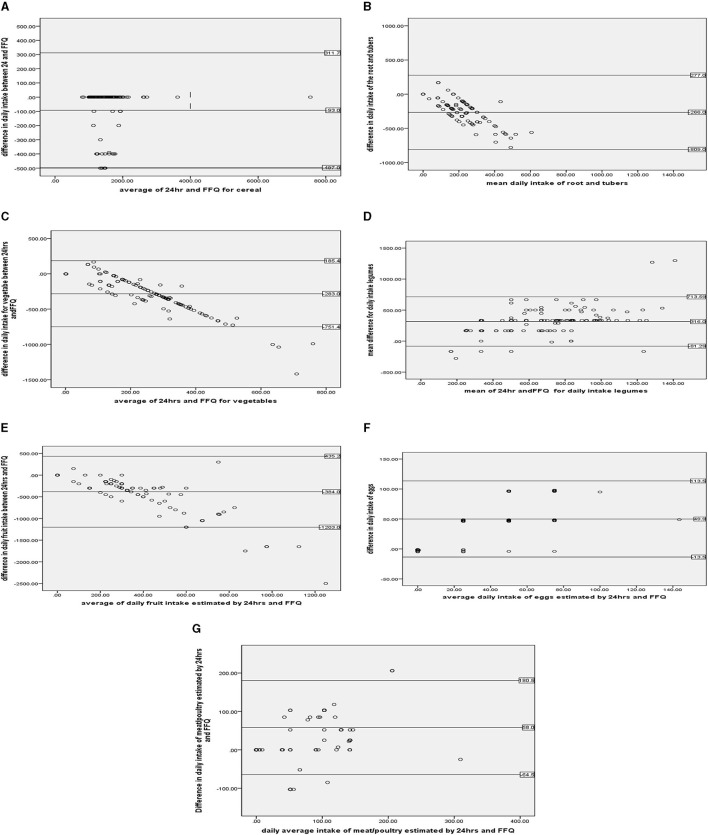
**(A–G)** Shows the Bland–Altman plots for food group intake. Data related to cereal, legumes, roots and tubers, vegetables, fruits, eggs, and meat/poultry/for Bland and Altman statistics.

The Figures 2A, B shows agreement of the two measurements within the 95% limit of agreement for cereal and its product within the difference range from 31 to 497. Similarly, for roots and tubers, the 95% limit of agreement for two measurement ranges was −809 to 277 (Figure 2).

The Figures 2C, D shows agreement between the two measurements within the 95% limit of agreement for vegetables within the difference range from −751 to 165.4. Similarly, for legumes, the 95% limit of agreement for two measurement ranges was −81 to 713.

The Figures 2E, F shows agreement of the two measurements within the 95% limit of agreement for fruits within the difference range from −1,203 to 384. Similarly, for eggs, the 95% limit of agreement for two measurement ranges was −13.5 to 113.5.

The Figure 2G shows agreement between the two measurements within the 95% limit of agreement for poultry and its products within the difference range from −64 to 180.5.

## Discussion

This study aimed to validate a food frequency questionnaire to assess the food intake of adults in the Ale district. The difference in score between two measurements was calculated, and a one-sample *t*-test was conducted to check whether the difference between two scores was not different from zero. Then, Bland–Altman plots were plotted to investigate the agreement between the two measures and how much the measures agreed with one another. Linear regression was conducted to check for proportional bias between the data points above the mean difference line. Then, the coefficient for the mean was checked for zero, and the null hypothesis was accepted, which stated that there was no trend difference in the data points above and below the mean difference line. The food frequency questionnaire overestimates the diary product and beverages compared to the 24-h dietary recall methods.

After checking for normality between the food intake estimated by the FFQ and 24-h dietary recall methods and found to be significant, the Wilcoxon signed test, which is an equivalent nonparametric test for paired samples, was used. The Wilcoxon signed test was conducted to determine whether there was a difference in the median score. The signed test showed the presence of a median difference between the two measurements in estimating the median daily intake of cereal and its products, vegetables and beverages. For legumes, roots and tubers, eggs, dairy products, fruits and meat/poultry showed good agreement between the two measures in estimating daily median intake. Compared with 24-h dietary recall methods, food frequency questionnaires tend to overestimate the median daily intake of vegetables and beverages. The overestimation of daily intake of vegetables and beverages might be due to overreporting of their consumption over the period of time, and this could also be due to a failure to understand the portion size assigned to the food groups. This finding is supported by a validation study conducted among adult community-dwelling older adults in a Mediterranean country, Lebanon (8). In contrast, food frequency questionnaires underestimate the daily intake of cereal and cereal products. This finding is in line with the findings of a previous study conducted on Iranian adolescents (4). This could be attributed to the recalling of past dietery consumption, which may be affected by the ability to memorize the size portion.

After our data failed to fulfill the assumptions of Pearson correlation with the significant Kolmogorov–Simonov normality test, we moved to the next step to check the assumptions of Spearman correlation. Then, after checking for monotonic correlations for the variables, the Spearman correlation coefficient was calculated to determine the strength of the relationship between the two measures. Spearman correlation coefficients between the 24-h food intake and the food frequency questionnaire ranged from 0.02 for vegetables to 0.85 for vegetables. This study revealed weak correlations (0.2–0.4) for beverages (*r* = 0.2), vegetables (*r* = 0.3), and roots/tubers (*r* = 0.34) and moderate correlations (0.4–0.7) for fruits (*r* = 0.43) and meat/poultry/fish (*r* = 0.05). A strong correlation (0.7–0.99) was found for dairy products (*r* = 0.73), eggs (*r* = 0.74) and vegetables (*r* = 0.85) between the two measurements. All food groups were significantly different except for beverages and meat/poultry. This moderate to strong correlation was in line with the results of validation studies among adolescents from different studies (5, 8, 17). This could be due to the use of triple 24-h dietary recall data, which can achieve an overall strong correlation by providing comprehensive and exhaustive food intake data. This study revealed a greater correlation for vegetables (*r* = 0.85). This can also be attributed to easy quantification of vegetables due to frequent consumption in the study area. The lower beverage correlation of *r* = 0.2 could be due to intentional underreporting of alcohol consumption over a longer period. This is supported by findings from studies among adults in Montreal (6, 16).

The proportion of individuals classified by the FFQ and the average of two 24-h dietary recalls into the same quartile ranged from 2% for beverages to 61.3% for legumes. This finding was higher than the findings of a similar validation study from Butajira among adults (7). This might be due to the use of the triple 24-h dietary recall method, which improved the categorization through approaching the values from the food frequency questionnaire. However, the proportion classified into opposite quartiles varied from 2% for cereal and cereal products to 24% for beverages. This finding contradicts the findings from validation studies conducted in older adults Iranian people, Mexico Egypt and the Lebanese (1, 4, 5, 10). This could be due to the overestimation of 24-h dietary intake of cereal and beverages compared to the overall consumption. The weighted kappa values ranged from good (wk = 0.61–0.80) for eggs (wk = 0.7) to very good (wk = 0.81–1) for legumes (wk = 0.9). This shows good agreement between the two measurements of food intake. This finding is comparable to the findings from validation studies conducted among Bangladesh and Lebanese populations (9, 14).

After conducting the one sample *t*-test for the difference between the two measures for each food group, we tested the hypothesis that the mean difference was not different from zero. We found that cereal, vegetable, meat/poultry, fruit, root/tubers, eggs and legumes were not significantly different. Then, Bland–Altman plots were constructed for these food groups. Finally, linear regression was conducted to check for proportional bias for these food groups, and the results were statistically nonsignificant. This shows the absence of proportional bias. The findings from the Bland–Altman plot showed poor agreement (wk < 0.5) to excellent agreement (wk >0.8) between the two measures. For food groups such as vegetables, legumes, and roots/tubers, the Bland–Altman plot showed excellent agreement between the two measurements (wk >0.8). This finding is supported by similar studies showing that most data points also exist within the limit of agreement (1, 8, 17, 19). Similarly, this study showed good agreement between the two measurements for cereal, eggs, dairy products and meat/poultry. This finding is supported by similar validation studies in Lebanon (8, 9, 13, 15, 20). In contrast, the findings of these measurements showed poor agreement for the fruits and beverages. This finding is supported by the findings from the meta-analysis, which showed poor agreement. However, the current study showed wider limits of agreement for fruits (−1,203, 435.2) and beverages (−0.84, 2.6) [compared to the findings from meta-analyses of fruits (−168, 668) and beverages (−0.31, −0.6)] (2, 5, 18). This might be due to the difference in the number of study participants involved in the meta-analysis.

In general, this study showed good agreement between these two measurements for the food intake of legumes, roots/tubers, vegetables, cereals, eggs, dairy products, and meat/poultry, whereas the FFQ overestimates fruit consumption (wk = 0.33) and underestimates that of beverages (wk = 0.2).

### Strengths of the study

- This study aimed to validate a food frequency questionnaire in Ethiopia, using the updated national dietary document. Key informants and group discussions were conducted to identify the local diet for the questionnaire. The questionnaire was pretested in the field during data collection training to ensure reliability and it was 1.038. The 24-h dietary recall test was conducted three times, on fasting days, working days, and weekends. A comprehensive statistical analysis was performed using methods such as Wilcoxon signed test, correlation coefficient, cross-classification, weighted kappa, and Bland-Altman statistics. The study's response rate was satisfactory, making it the first of its kind in the area.

### Limitations of the study

- The study found that recall bias could have been introduced due to the number of food groups consumed and the size of the food group. To reduce this, 24-h dietary recall was performed three times and portion size photos were distributed to each data collector. Selection bias might have been introduced due to the lack of stratification during the sampling procedure. Social desirability bias could have resulted in overreporting or underreporting of food group consumption.

## Conclusions

- In summary, the supporting individual-level validity was acceptable for food intake, as indicated by correlation coefficients and Bland–Altman plots.
- This study revealed that this food frequency questionnaire had good validity for capturing the intake of food groups such as vegetables, legumes, roots/tubers, cereal, dairy products and meat/poultry at both the individual and group levels. The interpretation of fruit intake requires caution.
- The FFQ should not be used to estimate beverages and has a positive proportional bias.
- Food frequency questionnaires can enable individuals to be ranked based on cereals, roots and tubers, fruits, vegetables, dairy product eggs, and meat/poultry.

### Recommendations

For Woreda culture and tourism bureau, agriculture bureau.

- The supply of different food groups should be improved based on the frequency of consumption.

For health care providers

- The FFQ was incorporated as a tool for studying and managing dietary-related diseases. This includes the prevention and control of noncommunicable diseases.

Researcher

- Future researchers are expected to conduct studies through the inclusion of energy and nutrient intake in the study area.
- Further research should also be conducted to assess the reliability of the food frequency questionnaires.

## Data Availability

The original contributions presented in the study are included in the article/supplementary material, further inquiries can be directed to the corresponding author.

## References

[1] Adeba A Tamiru D Belachew T. Healthy dietary practices and its' associated factors among adults of Nekemte dwellers, Oromia State, Western Ethiopia. Front Nutr. (2024) 10:1259024. 10.3389/fnut.2023.1259024 38328684 PMC10847308

[2] Malekahmadi M Naeini AA Shab-bidar S Feizi A Djazayery A. Development, validity, and reliability of a food frequency questionnaire for antioxidants in elderly Iranian people. J *Res Med Sci*. (2016) 21:14. 10.4103/1735-1995.178753 27904560 PMC5122246

[3] Tollosa DN Van Camp J Huybrechts I Huybregts L Van Loco J De Smet S. Validity and reproducibility of a food frequency questionnaire for dietary factors related to colorectal cancer. Nutrients. (2017) 9:1–17. 10.3390/nu9111257 29149033 PMC5707729

[4] Zack RM Irema K Kazonda P Leyna GH Liu E Gilbert S. Validity of a food frequency questionnaire to measure nutrient and food intake in Tanzania. Public Health Nutr. (2019) 21:2211–20. 10.1017/S1368980018000848 29656731 PMC6101256

[5] Ortega RM Perez-Rodrigo C Lopez-Sobaler AM. Métodos de evaluación de la ingesta actual: registro o diario dietético. Nutr Hosp. (2015) 31:38–45. 25719769 10.3305/nh.2015.31.sup3.8749

[6] Tabacchi G Filippi AR Amodio E Jemni M Bianco A Firenze A. A meta-analysis of the validity of FFQ targeted to adolescents. Public Health Nutr. (2015) 19:1168–83. 10.1017/S1368980015002505 26354204 PMC10270808

[7] Bedard D Shatenstein B Nadon S. Underreporting of energy intake from a self-administered food-frequency questionnaire completed by adults in Montreal. Public Health Nutr. (2004) 7:675–81. 10.1079/PHN2003578 15251058

[8] Regassa IF Endris BS Habtemariam E Hassen HY Ghebreyesus SH. Development and validation of food frequency questionnaire for food and nutrient intakes of adults in Butajira, Southern Ethiopia. J Nutr Sci. (2021) 10:1–13. 10.1017/jns.2021.94 34888036 PMC8635871

[9] Yaghi N Boulos C Baddoura R Abifadel M Yaghi C. Validity and reliability of a food frequency questionnaire for community dwelling older adults in a Mediterranean country: Lebanon. Nutr J. (2022) 21:1–13. 10.1186/s12937-022-00788-8 35717319 PMC9206140

[10] Shinozaki N Yuan X Murakami K Sasaki S. Development, validation and utilization of dish-based dietary assessment tools: a scoping review. Public Health Nutr. (2021) 24:223–42. 10.1017/S136898002000172X 32758321 PMC7808862

[11] Moghames P Hammami N Hwalla N Yazbeck N Shoaib H Nasreddine L. Validity and reliability of a food frequency questionnaire to estimate dietary intake among Lebanese children. Nutr J. (2016) 15:1–12. 10.1186/s12937-015-0121-1 26753989 PMC4709981

[12] Gibson RS Charrondiere UR Bell W. Measurement errors in dietary assessment using self-reported 24-hour recalls in low-income countries and strategies for their prevention. Adv Nutr. (2017) 8:980–91. 10.3945/an.117.016980 29141979 PMC5683000

[13] Gibson R Ferguson E. An Interactive 24-Hour Recall for Assessing the Adequacy of Iron and Zinc Intakes in Developing Countries. (2008). Available at: https://www.researchgate.net/publication/228556590

[14] Naska A Lagiou A Lagiou P. Dietary assessment methods in epidemiological research: current state of the art and future prospects. F1000Res. (2017) 6:1. 10.12688/f1000research.10703.1 28690835 PMC5482335

[15] Melaku YA Temesgen AM Deribew A Tessema GA Deribe K Sahle BW. The impact of dietary risk factors on the burden of noncommunicable diseases in ethiopia: findings from the global burden of disease study. Int J Behav Nutr Phys Act. (2013) 13:122. 10.1186/s12966-016-0447-x 27978839 PMC5159959

[16] Lin PD Bromage S Mostofa G Allen J Oken E Kile ML. Validation of a dish-based semiquantitative food questionnaire in rural Bangladesh. Nutrients. (2017) 9:49. 10.3390/nu9010049 28075369 PMC5295093

[17] MacIntosh DL Williams PL Hunter DJ Sampson LA Morris SC Willett WC. Evaluation of a food frequency questionnaire-food composition approach for estimating dietary intake of inorganic arsenic and methylmercury. Cancer Epidemiol Biomarkers Prev. (1997) 6:1043–50. 9419401

[18] Cade J Thompson R Burley V Warm D. Development, validation and utilization of food-frequency questionnaires – a review. Public Health Nutr. (2002) 5:567–87. 10.1079/PHN2001318 12186666

[19] Me H Schroeder D Rodri MM. Validation of a semiquantitative food-frequency questionnaire for use among adults in Guatemala. Public Health Nutr. (2002) 5:691–8. 10.1079/PHN2002333 12372164

[20] Boucher B Cotterchio M Kreiger N Nadalin V Block T Block G. Validity and reliability of the Block98 food-frequency questionnaire in a sample of Canadian women. Public Health Nutr. (2006) 9:84–93. 10.1079/PHN2005763 16480538

[21] Nasreddine L Naja F Arnaout S. Validation of a Food Frequency Questionnaire and A Spot Urine Sample for the Assessment of Dietary Sodium Intake in Lebanese Adults. Beirut: American University of Beirut (2016).

